# Supplementary material for: NAGS, CPS1, and SLC25A13 (Citrin) at the Crossroads of Arginine and Pyrimidines Metabolism in Tumor Cells
Source: Int J Mol Sci. 2023 Apr 4;24(7):6754. doi: 10.3390/ijms24076754 (PMC10094985; doi:10.3390/ijms24076754)
Supplement: Supplementary file 1 [file ijms-24-06754-s001.zip › ijms-2241907-supplementary/Supplementary_Tables_Figures/Supplementary_Figures.pdf]

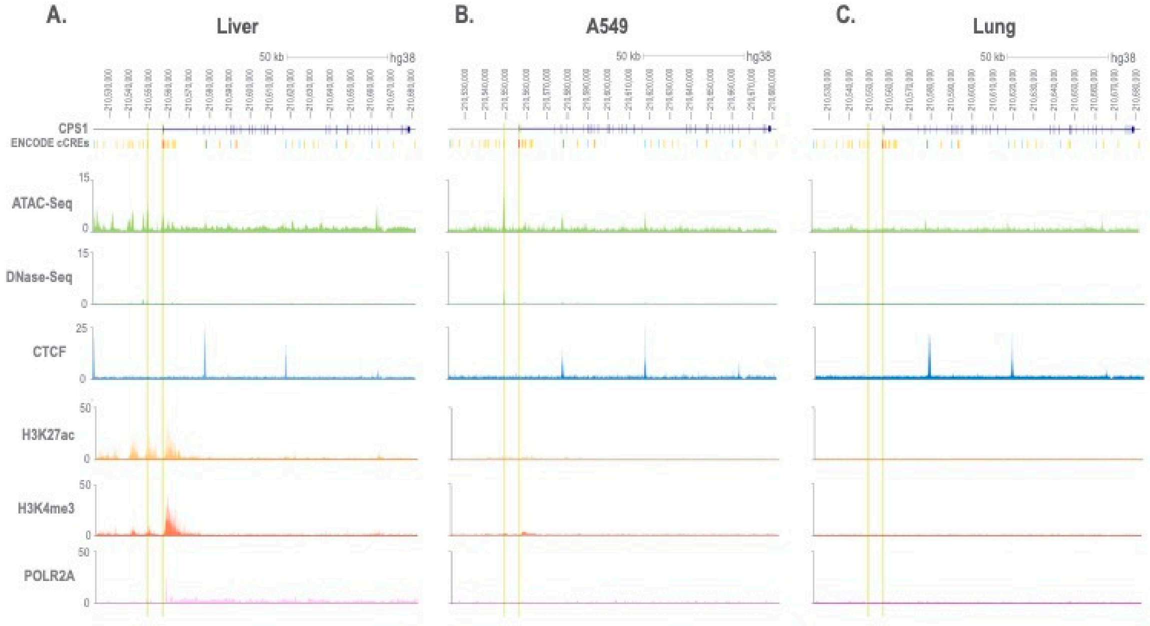

Figure S1. Epigenetic regulation of the *CPS1* locus in the liver tissue (A), A549 cell line (B) and lung tissue (C). Chromatin accessibility (ATAC-Seq and DNase-Seq), CTCF binding, acetylation of histone H3 lysine 27 (H3K27ac), tri-methylation of histone H3 lysine 4 (H3K4me3) and binding of the 2A subunit of RNA polymerase II (POLR2A) are shown for the genomic region: chr2: 210,522,000-210,680,000. Predicted cCREs: promoters – red, proximal enhancers – orange, distal enhancers – yellow, chromatin insulators – blue.

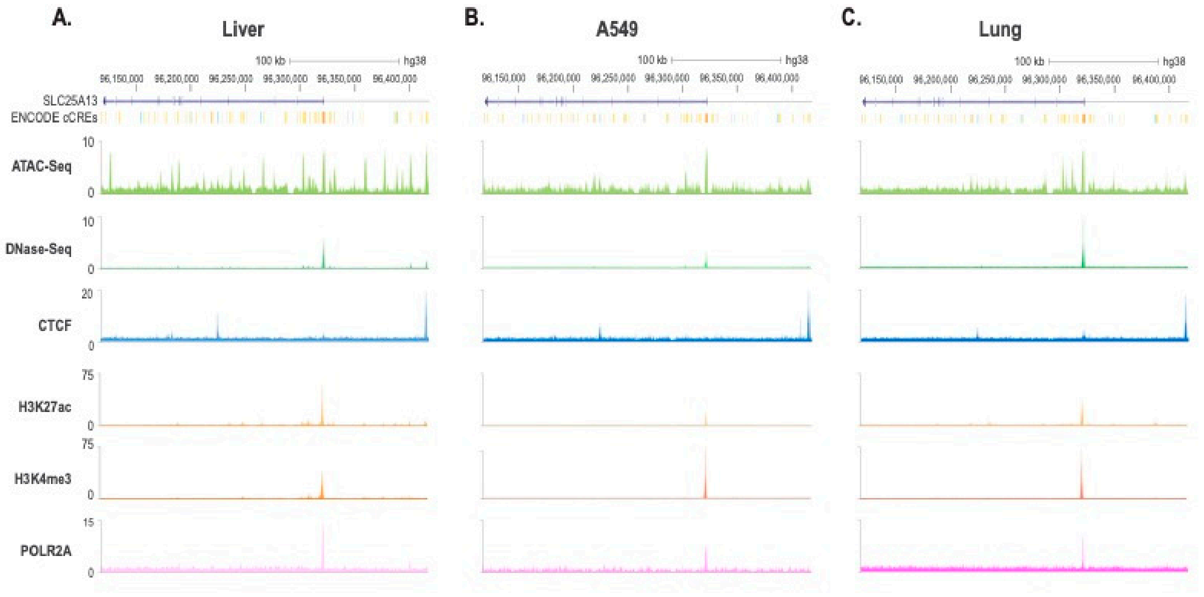

Figure S2. Epigenetic regulation of the *SLC25A13* (Citrin) locus in the liver tissue (A), A549 cell line (B) and lung tissue (C). Chromatin accessibility (ATAC-Seq and DNase-Seq), CTCF binding, acetylation of histone H3 lysine 27 (H3K27ac), tri-methylation of histone H3 lysine 4 (H3K4me3) and binding of the 2A subunit of RNA polymerase II (POLR2A) are shown for the genomic region: chr7:96,100,989-96,418,696. Predicted cCREs: promoters – red, proximal enhancers – orange, distal enhancers – yellow, chromatin insulators – blue.
